# Spatially-Resolved Eigenmode Decomposition of Red Blood Cells Membrane Fluctuations Questions the Role of ATP in Flickering

**DOI:** 10.1371/journal.pone.0040667

**Published:** 2012-08-10

**Authors:** Daniel Boss, Annick Hoffmann, Benjamin Rappaz, Christian Depeursinge, Pierre J. Magistretti, Dimitri Van de Ville, Pierre Marquet

**Affiliations:** 1 Brain Mind Institute, Ecole Polytechnique Fédérale de Lausanne, Lausanne, Vaud, Switzerland; 2 Advanced Photonics Laboratory, Ecole Polytechnique Fédérale de Lausanne, Lausanne, Vaud, Switzerland; 3 Centre de Neurosciences Psychiatriques, Département de psychiatrie DP-CHUV, Prilly-Lausanne, Vaud, Switzerland; 4 Institute of Bioengineering, Ecole Polytechnique Fédérale de Lausanne, Lausanne, Vaud, Switzerland; 5 Department of Radiology and Medical Informatics, University of Geneva, Geneva, Switzerland; Dalhousie University, Canada

## Abstract

Red blood cells (RBCs) present unique reversible shape deformability, essential for both function and survival, resulting notably in cell membrane fluctuations (CMF). These CMF have been subject of many studies in order to obtain a better understanding of these remarkable biomechanical membrane properties altered in some pathological states including blood diseases. In particular the discussion over the thermal or metabolic origin of the CMF has led in the past to a large number of investigations and modeling. However, the origin of the CMF is still debated. In this article, we present an analysis of the CMF of RBCs by combining digital holographic microscopy (DHM) with an orthogonal subspace decomposition of the imaging data. These subspace components can be reliably identified and quantified as the eigenmode basis of CMF that minimizes the deformation energy of the RBC structure. By fitting the observed fluctuation modes with a theoretical dynamic model, we find that the CMF are mainly governed by the bending elasticity of the membrane and that shear and tension elasticities have only a marginal influence on the membrane fluctations of the discocyte RBC. Further, our experiments show that the role of ATP as a driving force of CMF is questionable. ATP, however, seems to be required to maintain the unique biomechanical properties of the RBC membrane that lead to thermally excited CMF.

## Introduction

Throughout their lifespan of about 100–120 days, erythrocytes, while being carried throughout the vascular tree to deliver oxygen from the lungs to the tissues, are squeezed as they pass capillaries often smaller than the cell diameter. This ability can be attributed to the remarkable elastic properties of the membrane structure, which consists of a lipid bilayer that is coupled at specific binding sites to the underlying cytoskeleton. This membrane structure exhibits a high resistance to stretch ensuring that no leakage through the lipid bilayer occurs, whereas its low resistance to bending and shear allows the cell to undergo easily shape changes when passing through small capillaries. As a consequence of these elastic properties, RBCs show spontaneous cell membrane fluctuations (CMF), often named flickering, that can be observed under the microscope. This phenomenon has been the subject of considerable scientific interest in the past. One reason is certainly that CMF provide information on the elastic properties of the RBC membrane structure and might help in the diagnosis or understanding of diseases affecting the RBC. Different views on RBC flickering have been at the origin of research approaches. Brochard and Lennon [1] were among the first to describe quantitatively CMF characteristics. In their theoretical model of the flickering phenomenon, CMF are explained as thermally excited undulations of the cell membrane that are mainly governed by the bending elasticity of the membrane. Using the theoretical model of Brochard and Lennon, a bending modulus of 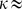
(2–7)e-20 J was determined in a short wavelength regime where the approximation of a flat membrane is valid [1]–[3]. Strey et al. [4] estimated the bending and shear modulus of erythrocytes in a long wavelength regime based on an eigenmode decomposition of CMF. Contrary to the Brochard and Lennon approach, their theoretical model accounts for a closed cell surface with non-zero equilibrium curvature. A bending modulus of 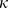 = 4e-19 J was measured in this long wavelength regime. A common result in the above mentioned reports is that shear deformation energy in flickering is negligible; i.e., the shear elasticity of the cytoskeleton is not sensed by the membrane flickering motions in the discocyte RBC. More refined elastic theories that take into account the bilayer-cytoskeleton interactions during flickering were proposed recently [5], [6]. It is noteworthy that the above mentioned estimates of the elastic properties rely on the hypothesis that the driving force of fluctuations is purely thermal. Other authors, however, observed a CMF decrease upon ATP depletion, supporting the view that CMF have also a metabolic origin [7]–[10]. Levin et al. [7] suggested that an ATP dependent mechano-chemical dynamic assembly of the cytoskeleton could enhance CMF. In their view of flickering, the cytoskeleton can exert an active, ATP-consuming excitation of the bilayer. A similar view was shared more recently by Park et al. [10]. According to these authors, dynamic binding and unbinding of the cytoskeleton to the bilayer in the presence of ATP can increase CMF. In contrast, Evans et al. [11], and Szekely et al. [12] found a lack of ATP dependence on the CMF in the discocyte RBCs.

In this report we show that the CMF of the normal discocyte RBC, as measured with digital holographic microscopy (DHM), can be decomposed into spatially-resolved fluctuation eigenmodes. DHM and other similar quantitative phase microscopy techniques have only recently been applied to study the CMF in RBCs [10], [13], [14]. The instantaneous recording of the CMF on the cell surface with nanometric sensitivity along the optical axis and at high temporal and spatial resolution has several advantages over other techniques where CMF can be observed only at the rim of the cell or at single points on the cell surface. DHM is particularly well suited to explore cell dynamics [15], [16] and allows for an accurate spatial analysis of the CMF [13]. We proceed the imaging data using principal component analysis (PCA) [17] to characterize the experimental CMF measurements by providing a series of spatial maps corresponding to a basis of eigenvectors that maximize the spatial variance. These experimental PCA modes are compared to deformation eigenmodes from a theoretical model of the elastic cell structure. As model parameters we include only the bending elasticity of the membrane and the stationary RBC shape that arises from bending energy minimization. However, different bending elasticity concepts have emerged over the years. Using the spontaneous curvature (SC) bending elasticity, Helfrich [18], [19] was able to predict various cell shapes of the RBC that can be found in nature. The bilayer coupling hypothesis (BCH), originally introduced by Sheetz et al. [20], is a slightly different curvature concept, but leads to the same stationary RBC shapes. Peterson predicted the spatial distribution of CMF amplitudes on the RBC cell surface based on the minimization of these two bending energies [21], [22]. Our model, which relies partially on the work of Peterson, allows to reproduce fluctuation eigenmodes whose spatial deformations and fluctuation amplitudes are in good agreement with the observed PCA modes. To gain a better understanding of the ATP dependency of CMF amplitudes and metabolic implications, the proposed method is applied to RBCs presenting various levels of ATP depletion. Our finding that the CMF decrease does not directly correlate with the intracellular ATP concentration during depletion questions the role of ATP as a metabolic substrate that drives CMF.

## Results and Discussion

### PCA mode decomposition of CMF based on DHM measurements

**Figure 1:**
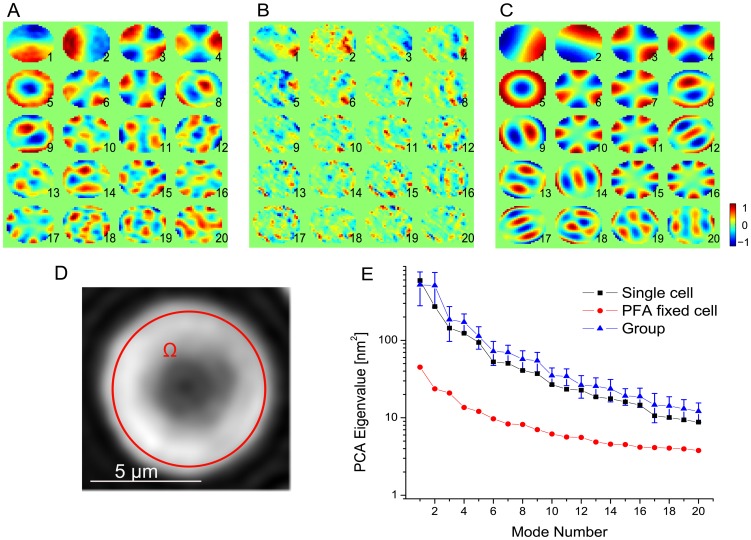
PCA Results. Normalized PCA modes (A) of a single cell (B) of a paraformaldehyde fixed cell (C) of a group of n = 198 cells. The spatial distribution of the normalized mode amplitude is color coded. (D) Phase image of erythrocyte with typical ROI for PCA analysis (E) Eigenvalues of PCA modes of A,B,C (Log Scale). Standard deviations of the values 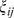 are indicated by error bars.

DHM provides instantaneous quantitative phase measurements in time and space from which the cellular thickness 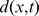 can be deduced at any point on the cellular surface. CMF are assessed locally on the cell surface in a region of interest (ROI) (cf. Fig. 1D) by measuring the thickness fluctuation around the local average cell thickness. Using PCA, we are able to decompose the CMF measurements into (orthogonal) spatial maps and associated temporally coherent fluctuations. Figure 1A shows the first 20 PCA modes 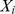 of a single cell remapped to the ROI and ordered by decreasing eigenvalue 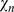. The associated eigenvalue 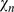 is a measure of the temporal variance of the component (*see* *Materials and Methods*). PCA modes can be understood as the preferential spatial deformations of the cell membrane that occur during flickering. Although PCA does not impose any spatial regularity, the obtained PCA modes show spatially well-defined patterns for the CMF of a single cell already. As can be expected for CMF that are governed mainly by the bending elasticity of the membrane, modes with large spatial wavelength exhibit the highest fluctuation amplitudes. In a control experiment the same type of measurements were obtained with paraformaldehyde fixed cells, where corresponding PCA modes showed only noisy patterns and associated eigenvalues 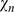 that were about an order of magnitude lower compared to unfixed cells (cf. 1B,E). Thus the cross-linkage of proteins in the membrane with paraformaldehyde strongly inhibits CMF and no mode decomposition is detected with our approach. Using group-level PCA, the population eigenmodes for n = 198 erythrocytes were retrieved. The inherent spatial eigenmode decomposition of CMF becomes obvious (cf. Fig. 1C). Even for a much smaller number of cells (n
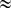
20), the same spatially well-defined PCA modes are retrieved and the first 16 components are revealed with no change in eigenvalue order. Most PCA modes have a degeneracy of two, meaning that two linearly independent PCA modes represent the same physical deformation. This property relates to the steerability of the PCA modes; e.g., the first mode can be turned along any orientation by a proper linear combination of itself and the second mode. The eigenvalues of these degenerated PCA modes are statistically equal for the group-level PCA (cf. Fig. 1E). We observed that for some single-cell recordings the eigenvalues of degenerated modes remained different, even when increasing the observation time, suggesting asymmetric shape or cell attachment that favors one (degenerate) mode versus the other. For the higher order PCA modes of the group one observes that the spatial pattern of the components is less influenced by the geometry of the cell and planar modes become predominant (*see Figure S1*).

### Eigenmode decomposition based on bending deformation energy

**Figure 2:**
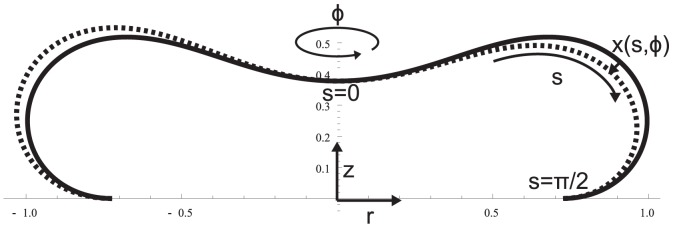
RBC Equilibrium Shape. Straigth line: Equilibrium shape that arises from bending energy minimization (see Text S1) and which was used in the calculations to derive eigenmodes of the bending deformation energies depicted in Figure 3B,C. Dashed line: Shape of deformed cell.

To evaluate whether the observed PCA modes can be understood as thermally excited fluctuation eigenmodes that are, just as it is the case for the equilibrium cell shape, subject to bending energy minimization, we proceed as follows. A discocyte cell shape that minimizes the SC and BCH bending energy 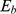 is identified. The approach presented in Seifert et al. [23] was therefore adopted (*see Text S1*). An asymmetric cell shape that attaches to the polyornithine coated coverslip was modeled. Our hypothesis is that the regions where the cell membrane attaches to the coverslip show strongly diminished CMF and are therefore not considered as a part of the undulating membrane (see Fig. 2). CMF of the equilibrium cell shape are described by a deformation function 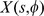 transporting the membrane in the direction normal to the cell surface. Such a deformation function can always be decomposed onto fluctuation eigenmodes 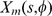 that are subject to bending energy minimization. For small deformations it is sufficient to minimize a second order energy functional in deformation coordinates (bending deformation energy) that describes the bending energy of the cell shape around its stationary configuration. We considered three different bending deformation energies, namely the SC, BCH and Laplace deformation energy (*see* *Materials and Methods*) and derived for each case normalized energy eigenmodes 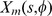 with associated bending deformation energies 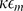.

### Comparison of theoretical energy eigenmodes and experimental PCA modes

Based on the equipartition theorem which states that in thermal equilibrium each degree of freedom has an average thermal energy of 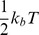 (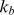 Boltzmann constant, 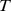 temperature in kelvin), we can predict a mean square fluctuation amplitude 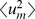 for an eigenmode in a cell with radius R and bending modulus 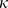 of


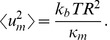
(1)

**Figure 3:**
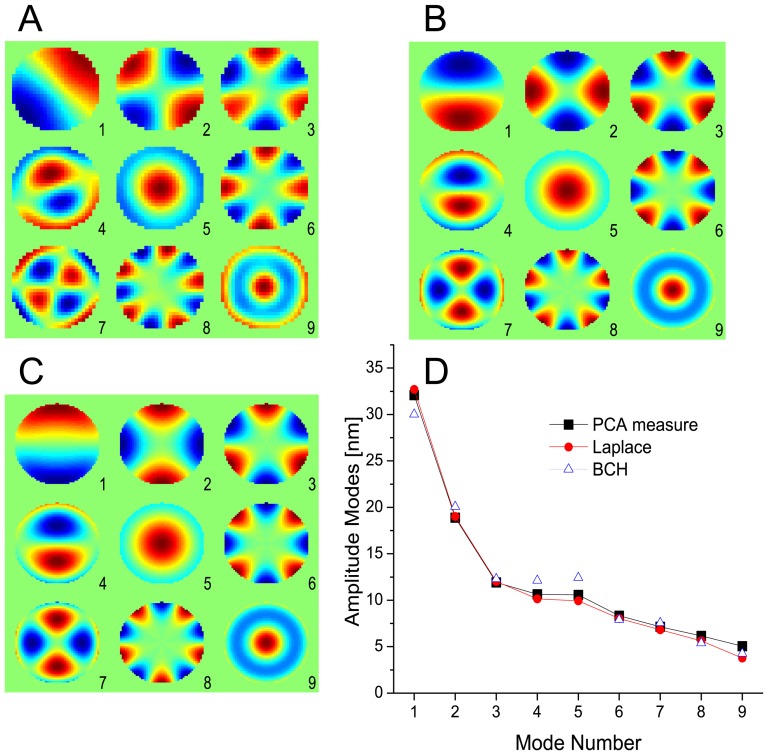
Experimental and Theoretical Comparison. (A) Degeneracy free representation of PCA modes. The separable modes 7 (degeneracy of 2) and 9 in this representation are obtained from an appropriate linear combination of the degenerate modes 12,13 and 14 of Figure 1C. Both representations are equivalent, because they span the same subspace. (B) Eigenmodes that minimize Laplace deformation energy shown in the ROI. Eigenmodes are plotted as functions 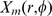 (C) Eigenmodes that minimize BCH Deformation Energy shown only in the ROI (D) Comparison of theoretical and measured fluctuation amplitudes.

The validity of this approach, especially the independence of mean fluctuation amplitudes on intracellular and extracellular viscosity has been suggested by Brochard and Lennon [1]. If the equipartition theorem holds, it gives a direct link between PCA modes that maximize temporal variance of CMF data and theoretically eigenmodes that minimize the deformation energy of small deformations (see *Material and Methods* for details on how the theoretically estimated mean square fluctuation amplitude can be compared to measured PCA mode variances). In Figure 3A, a degeneracy free and separable representation of the first 9 PCA modes ordered by decreasing amplitude is shown. This representation allows comparing the PCA modes to the theoretically calculated bending deformation eigenmodes. Figures 3B and C show the theoretical eigenmodes obtained when the simple Laplace, and the BCH deformation energy, respectively, are used for the derivation. Energy eigenmodes are shown only in the ROI as they would appear in a PCA experiment. For the Laplace bending energy, the eigenmodes exhibit a spatial distribution similar to the experimental PCA modes. In addition, the amplitudes of the eigenmodes calculated with Eq. 12 and the measured amplitudes of the PCA modes are similarly distributed. Consequently, the amplitude distribution of the eigenmodes can be matched to the PCA distribution when fitting with an appropriate value of the bending modulus (cf. Fig. 3D). It follows that the mean bending modulus of the group can be determined. In order to estimate a bending modulus for individual cells, we projected each cell onto the PCA modes of the group to evaluate the mode variances 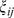. For each single cell, the bending modulus is then determined by fitting the theoretical eigenmode amplitudes to the the values 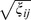. A mean bending modulus of 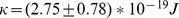 was measured over n = 198 cell sample when the Laplace bending deformation energy is considered. This value is in good agreement with previous measurements in this long wavelength regime of flickering [4], [24], [25]. This is a surpising result considering that Laplace bending energy is usually postulated for flat, relaxed membranes. A possible physical interpretation of this result could be that the membrane can over time locally relax the bending induced strain. One could imagine a spontaneous curvature of the biliayer that is locally adapted to match the real curvature of the bilayer. Such a locally adapted spontaneous curvature would make the Laplace energy the natural description for the bending energy of small deformations. What could be a possible mechanism that would allow a relaxation of the bending energy? Bending of the bilayer, for instance towards the inner layer, is presumably accompanied with an increased lipid density on the inner layer (through compression strain) and a decreased lipid density on the outer layer (through dilation strain). Because of the fluid properties of the membrane, relaxation of compression and dilation strains in each monolayer is then likely to occur via lateral, intramembrane diffusion of lipids towards regions of lower density [26], [27]. If the lipid flow is too slow to equilibrate lipid densities in the monolayers during the decay time of bending eigemodes, then the thermally excited shape fluctuations will not be relaxed with respect to the bilayer bending energy, and a simple Laplacian term for the bending energy of small deformations might be a good approximation.

However, also eigenmode shapes and amplitudes for the BCH energy, which is a more rigourous implementation of the bending elasticity concept, are still in good agreement with the PCA results. For the SC deformation energy, the theoretical evaluation do not yield such a good match on the measures. One mode is even unstable, i.e. the evaluated energy is negative (*see Table S1 and Figure S3*). Maybe an appropriate choice of the value of 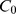 could prevent this instabilities. Similar, however, to what we observe, it has already been stated in reference [24] that the BCH energy is more appropiate than the SC energy to describe CMF. Interestingly, Lim et al. [28] showed that the stomatocyte-discocyte-echinocyte shape transition can be reproduced with the BCH model upon variation of the surface area difference 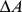. Thus stationary shapes, shape transitions and CMF all support evidence for the BCH. This general validity seems to make the BCH a suitable model to describe the membrane mechanics in the RBC. However, a small discrepancy between model and experimental measurements exists. Indeed an accurate analysis of the modes in figures 3A, 3B shows similar patterns but with a minute radial scale difference. Practically, the pattern of the shown theoretical eigenmodes spreads over an area on the RBC surface slightly larger than the corresponding PCA modes restricted to the area of the ROI (see Figure S4A,B). This difference might arise from an inadequate equilibrium shape of the model. In particular, the imposed boundary constraints cannot be verified experimentally. To assess the influence of the equilibrium shape and boundary constraints, we evaluated the Laplace energy eigenmodes and associated mode amplitudes for two slightly different equilibrium shapes. A good match of the evaluated eigenmode amplitudes with the PCA mode amplitudes is preserved, while the discrepancy in spatial localization of PCA modes and theoretical eigenmodes on the cell surface is reduced, altough not completely eliminated for every mode. Especially for modes with azimuthal mode number m = 0,2, the discrepancy is maintained (*see Figure S5*). Additionaly, the absence of a shear elasticity in the model might also contribute to this small discrepancy. In fact, Peterson [21] showed that an increased shear elasticity of the membrane tends to shift the position corresponding to maximal CMF amplitudes towards the center of the cell. When comparing measured CMF amplitude distribution on the RBC surface with the theoretical CMF amplitudes derived from the evaluated eigenmodes, we find good agreement between model and measurement, with CMF amplitudes that peak at the thickest point of the cell (*see Figure S4A,B*). Again this seems to suggest that shear elasticity of the membrane does not substantially influence CMF. The same evidence has been reported before by several authors [1], [3], [24], [25]. Other authors, however, reported on a non-negligible influence of membrane shear elasticity on the CMF [29], [30]. These different conclusions on the influence of shear elasticity on flickering presumably originate from the different theoretical models that are used to fit the elastic moduli. With regard to the applied theoretical models, we consider it important that models take into account the equilibrium shape, as the measured PCA modes show how strong CMF are influenced by the equilibrium shape of the RBC.

The investigated PCA modes and inferred bending modulus describe the membrane fluctuations in a long wavelength regime. In a short wavelength regime, however, a bending modulus approximately ten fold lower has been measured [1]–[3]. Indeed, when the mean square fluctuation amplitudes 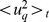 of planar modes are expressed in terms of spatial wave vector 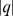, we observe, in a short wavelength regime, an eightfold increase of the fluctuation amplitudes when compared to the theoretically expected amplitudes for pure bending modes calculated with a bending modulus value 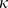 measured in the long wavelength regime (*see Figure S2*). It has been argued that this higher apparent bending modulus of the long wavelength regime compared to the short wavelength one, can be attributed to fluctuations of coupled fluid and solid membranes, i.e. bilayer and cytoskeleton [6]. A similar result has been reported recently by Park et al. [29] who measure a low bending stiffness also in the long wavelength regime, but include shear and area compressibility modulus to accomodate measurements and applied theoretical model. Thus the higher apparent bending modulus in the long wavelength regime would not only comprehend the bending modulus of the bilayer, but include terms related to the cytoskeleton and its interaction with the bilayer. If so, it is then surprising that the measured PCA modes can be properly described by a pure bending deformation energy model, which neglects the shear modulus of the cytoskeleton.

### CMF in ATP depleted RBCs

**Figure 4:**
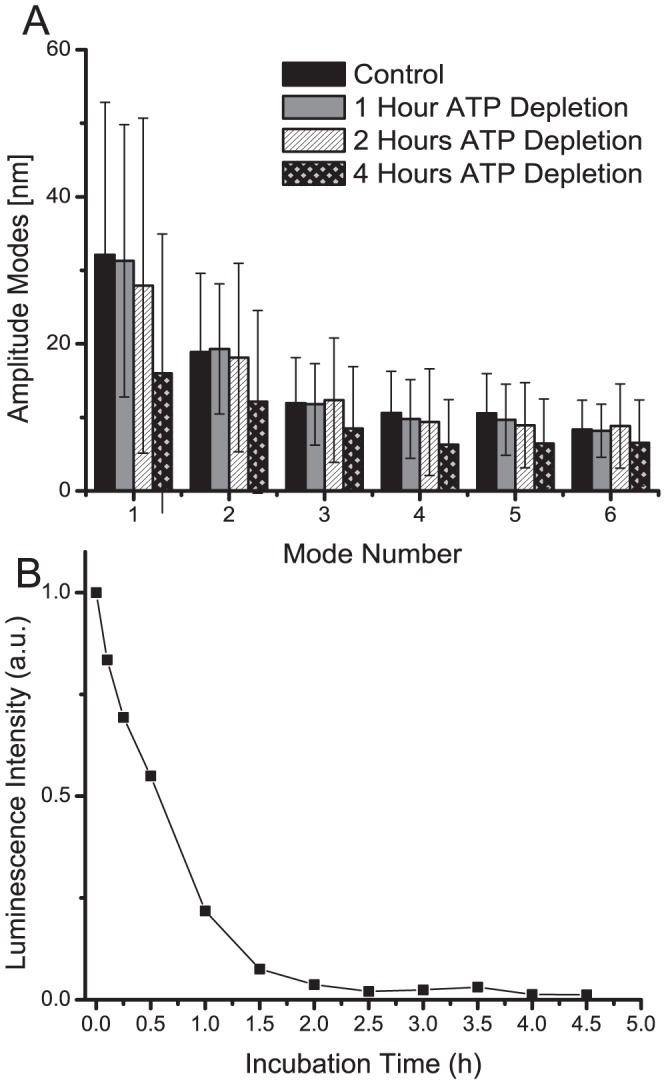
ATP Depletion in RBC. (A) Fluctuation amplitudes of the first six PCA modes in a population of n = 198 (no ATP depletion), 43 (1 h ATP depletion), 47 (2 h ATP depletion), 36 (4 h ATP depletion) cells. (B) Assessment of intracellular ATP level at different instants of incubation time in depletion medium through the measurement of chemiluminescence intensity of luciferin luciferase assay in RBC cell solution.

Bending energy eigenmodes whose amplitudes can be predicted based on the equipartition theorem seem to be in contradiction with active ATP-consuming mechanisms. Therefore, we applied the PCA method to investigate CMF in RBCs with various levels of ATP depletion. After 1 hour of incubation in the depletion medium, the equilibrium shape of all the RBCs in the population still remained biconcave. After 2 h of incubation the onset of echinocyte formation with small spicule protrusions on the cell rim was observed in some cells of the population, whereas after 4 h of incubation nearly all cells were spiculated. Fig. 4A shows the respective mean fluctuation amplitudes of the first 6 bending modes depicted in Fig. 3A. To assess the dynamics of the ATP depletion protocol, intracellular ATP levels were measured via luciferase chemiluminescence, whose emitted intensity is proportional to ATP concentration [31] (cf. Fig. 4B). A significant fluctuation amplitude decrease after 4 h of ATP depletion is observed for every PCA mode in a population of n = 36 cells. Interestingly, after 4 h a subpopulation of cells did not show any spatially coherent fluctuations, i.e. the single cell PCA modes were not spatially well-defined and also the temporal variances 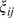 of the group PCA mode projections were nearly zero. Alteration of the membrane mechanical properties resulting from a strong binding of the cytoskeleton to the bilayer and/or a rigidification of the cytoskeleton could adequately explain such a result. Often such an increase in membrane rigidity upon ATP depletion is attributed to the dephosphorylation of the skeletal protein 4.1R that causes a reinforced binding to the transmembrane protein Glycophorin C and the actin filament at the junction of the spectrin tetramer in the cytoskeleton [10], [32], [33]. A further ATP dependent factor that might impact on CMF is the maintainance of lipid asymmetry in the bilayer via ATP consuming translocase activity (flippases and floppases). The phospholipid distribution among the leaflets has been show to modulate RBC membrane elasticity through altered spectrin-lipid interactions [34]. A possible mechanism is for instance the glycation of membrane-associated spectrin after disruption of lipid asymmetry through ATP depletion, which has been shown to reduce membrane deformability [35].

In contrast, an ATP-powered mechanism that would transfer mechanical energy to the bilayer and act as driving force of CMF as has been proposed by several authors [8]–[10], [33] should lead to fluctuation amplitudes that correlate with the intracellular ATP concentration. However, after 1 h of incubation in the depletion medium, where there are no significant changes of RBC cell shape or mode fluctuation amplitudes, the intracellular ATP concentration is reduced to 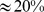 of the initial level. If ATP supplies energy to drive CMF, a drastic decrease in ATP should be reflected in decreased CMF. The observation that the intracellular ATP concentration is not correlated to CMF amplitudes and RBC shape has also been stated more recently by Szekely et al. [12]. As ATP plays certainly an important role in the maintenance of the mechanical properties of the composite membrane, our results suggests that ATP does not act as an active driving force of CMF. An additional argument is that the mean fluctuation amplitudes of the eigenmodes respect thermal equilibrium theory. However, this is not a definitive proof of the thermal origin of CMF, since it has been assumed that it is valid to model the effects of ATP by an increased effective temperature and preserve in this way thermal equilibrium [9]. However, one would expect in such a case a direct relation between CMF amplitudes and intracellular ATP concentration. In our view, the decrease of CMF after complete ATP depletion arises from the changed mechanical properties of the bilayer-cytoskeleton complex. The chemical processes that cause these mechanical changes are not yet fully understood. Similar to what has been suggested for ATP-mediated cell shape changes [36], [37], it seems that intracellular ATP does not directly determine CMF, but is among other factors responsible for the maintenance of the biomechanical properties of the cellular membrane.

### Conclusion

We have shown that the PCA which decomposes the experimental CMF measurements based only on a variance maximization criterium allows to identify spatially well-defined modes over the RBC surface. These PCA modes were confronted with a theoretical eigenmode decomposition of CMF which results from a quantization of the membrane deformation energy for fluctuations constrained by boundary conditions. Practically, considering only the bending deformation energy of the cell membrane around its stationary configuration has permitted to obtain eigenmodes presenting spatial patterns in good agreement with the PCA modes. In addition, the amplitude distribution of the eigenmodes calculated according to the equipartition theorem is similar to the amplitude distribution of the measured PCA modes. Consequently, matching the eigenmode amplitude distribution to the PCA amplitude distribution has allowed to determine the value of bending modulus 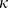. Experiments aimed at exploring the role played by ATP seem to favor a view in which CMF of erythrocytes are the result of thermal agitation governed mainly by the bending resistance of the cell membrane, whose mechanical properties are maintained by the presence of the intracellular ATP. Our approach which allows to characterize the CMF represents a useful tool to detect subtle changes in the mechanical properties of the RBC membrane.

## Materials and Methods

### Ethics statement

Blood withdrawal from healthy laboratory personnel was ethically approved by the Commission cantonale (VD) de la recherche sur l'être humain, Sous-Commission III, Rue César-Roux 19, 1005 Lausanne on january 16th 2012.

### Cell preparation

100–150 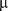
l of blood was drawn by fingerpick, collected and diluted at a ratio of 1∶10 (v/v) in cold HEP buffer (15 mM HEPES pH 7.4, NaCl 130 mM, KCl 5.4 mM and 10 mM glucose). Blood cells were sedimented at 200 g, 4

C for 10 min and buffy coat was gently removed. RBCs were washed twice in HEP buffer (1000 g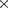)2 min at 4

C). Finally, erythrocytes were suspended in HEPA buffer (15 mM HEPES pH 7.4, 130 mM NaCl, 5.4 mM KCl, 10 mM glucose, 1 mM CaCl2, 0.5 mM MgCl2 and 1 mg/ml bovine serum albumin) at 0.2% hematocrit. 4 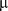
l of the erythrocyte suspension were diluted to 150 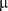
l of HEPA buffer and introduced into the experimental chamber, consisting of two cover slips separated by spacers 1.2 mm thick. The bottom coverslip was coated with polyornithine to ensure that cells adhere to the coverslip surface. This suppresses translational movements of the cells during acquisition and diminishes CMF of the attached cell membrane. Cells were incubated for 30 min at a temperature of 37

C before mounting the chamber on the DHM stage. All experiments were conducted at room temperature (22

C) and only cells displaying a normal discocyte shape were selected for analysis. All the experimental work reported in this paper relied only on blood samples taken from the co-authors DB and AH.

#### Irreversible ATP depletion in RBCs

RBCs were diluted in a glucose free HEPA buffer containing inosine, which consumes ATP, and iodoacetamide, that inhibits glycolysis. (10 mM HEPES, 120 mM NaCl, 5.4 mM KCl, 1.8 mM CaCl2, 0.8 mM MgCl2, 0.9 mM NaH2P04, 6 mM iodoacetamide, 10 mM inosine, 1 mg/ml bovine serum albumin) CMF measurements were conducted at room temperature after 1 h,2 h and 4 h of incubation at 37

C.

#### Measurement of ATP levels during depletion

Intracellular ATP content was assessed via the chemiluminescence intensity of a luciferin-luciferase based assay kit purchased from Promega (CellTiter-Glo Luminescence Cell Viability Assay). Luminescence intensity measurements were performed on a Tecan Safire well plate reader.

### DHM

Holograms were acquired on a transmission DHM setup (DHM T1000TM, LyncéeTec SA, Lausanne, Switzerland) as described in [15]. Briefly, the experimental setup is a modified Mach-Zehnder configuration with a laser diode source (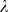 = 683 nm). The laser beam is split into a reference wave R and an object wave O. The latter is diffracted by the specimen, magnified by a 40X/0.75 NA microscope objective and interferes, in an off-axis geometry, with the reference wave to produce the hologram intensity recorded by the CCD camera. The reconstruction of the object wavefront is achieved through a numerical re-illumination of the hologram by a digital computed reference wave. The algorithm used for the wavefront reconstruction and aberration compensation has been described in [38]. Along the optical axis a nanometric sensitivity in cell height variations is achieved; lateraly DHM shows a diffraction limited resolution. Holograms of 256×256 pixels are acquired at a rate of 25 Hz from which phase images are reconstructed with custom-made software from LyncéeTec SA.

### PCA decomposition of CMF

As a first pre-processing step to PCA, the measured phase shift 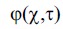 is converted into cellular thickness. Under the assumption of a constant cellular index of refraction this relation is given by


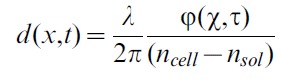
(2)

A mean cellular refractive index of 1.394, calculated by the same microscopic technique [39] and a refractive index of the HEPA solution of 1.3334 was therefore used. The assumption of a constant intracellular refractive index within the RBC volume is motivated by the fact that in the human RBC, lacking intracellular organelles and nucleus, the high refractive index is primarily due to the hemoglobin content that we suppose equally distributed within the cell. As a further preprocessing step, RBC images were aligned with an image registration algorithm [40] to ensure that local thickness variations were not due to a translational movement of the cell. A region of interest (ROI) (cf. Fig. 1D) is defined on the RBC that stretches slightly beyond the position of maximal thickness, but excludes the borders of the erythrocyte, since in these regions the cell surface shows high inclination with respect to the x–y plane (cf. Fig. 1D and Figure S4). These inclined border regions are not included because a small horizontal movement of the membrane translates into a strong axial displacement. The local thickness fluctuation amplitude u(x,t) for each pixel contained in the ROI is obtained by subtracting the temporal mean from the current thickness.


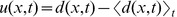
(3)

Because of the attachment of the lower RBC surface to the coverslip, we interpret the measured thickness fluctuations essentially as CMF of the upper (concave) RBC surface. PCA provides an orthogonal linear transformation that maps input data such that subsequent components maximize variance. PCA was applied to the spatial dimension of RBC fluctuations. Fluctuation amplitudes are stored in a matrix U,


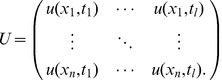
(4)

where l = 1000 images acquired over 40 s of a single cell region containing n pixels serve as input for the matrix U. By diagonalizing the covariance matrix 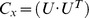, a spatial decomposition of the RBC membrane fluctuations that maximizes the variance (eigenvalue) 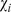 of the PCA mode (eigenvector) 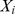 while cancelling out the covariance between modes is obtained. PCA modes 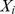 were ordered according to the importance of the associated variance 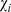.

#### Group PCA analysis

The method presented in the preceding paragraph restricts the analysis to a single cell. It is, however, important to extend the method to a larger number of cells and find the preferential PCA modes of a population. Therefore, RBC phase images are first reinterpolated to match the contour of a circular model cell. Resizing is performed with cubic spline interpolation. Artifacts of the interpolation procedure are estimated to be negligible, as PCA analysis results (eigenvalues and eigenmodes) on a single cell level are not altered by the cubic spline interpolation. The spatial covariance matrix of the group is obtained by summing up the covariance matrices of each cell,


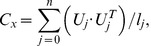
(5)

where n denotes the number of cells in the group and 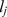 the number of acquired images of cell j. Diagonalization leads then again to the PCA modes. To assess the temporal evolution of a mode X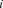 for a cell j we project the matrix U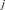 onto X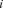 to form a vector u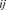 that holds the time course of the mode. The squared norm 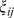 of this vector, i.e the temporal variance of the mode projection, can be compared to the eigenvalue 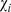 to evaluate the contribution of the cell to the PCA mode.

### Determination of bending deformation energy eigenmodes

An eigenmode that transports the membrane in a direction normal to the cell surface at each point 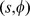 of the undulating membrane is described by a separable ansatz 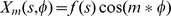, with m denoting the azimuthal number (cf. Fig. 2). In-plane-motions are not considered in the model because shape preserving displacements of the membrane do not alter bending energies. To determine the energy of a theoretical eigenmode 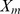 one can expand the bending energy 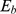 about its minimum up to second order with respect to a normal motion 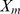. The first order term vanishes by stationarity and the second order term, the bending deformation energy, is a quadratic form in deformation coordinates as follows


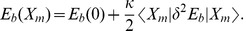
(6)

In this definition, the expression 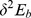 is a unitless measure of the energy cost of a deformation 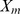 depending entirely on the cell geometry. In the case of the SC and BCH bending energies, Peterson [21], [22] derived explicit expressions for the bending deformation energy 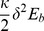 of normal motions 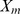. Further, another quadratic form referenced as Laplace deformation energy was investigated:


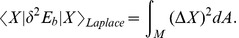
(7)

The Laplace deformation energy corresponds to the second order bending energy expansion for a flat, infinite, membrane. As such it must be considered as an ad-hoc approach when describing the bending deformation energy of a closed RBC cell shape.

To find an energy eigenmode 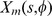 that minimzes the quadratic bending deformation energy 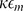, the Ritz method is applied. Following the Ritz method, we express the energy eigenmode 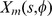 that minimzes a quadratic energy functional as a linear combination of basis functions 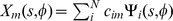 that all meet imposed constraints. In the present case, these constraints are 
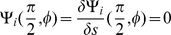
, i.e. the deformation function and its derivate vanish where the cell attaches to the coverslip. Additionaly, for modes with azimuthal number m = 0, one must take care that the cell does not change its initial volume in a deformation. Linear combinations of sinusoidal functions that respect the constraints are found as a convenient basis of functions 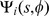. The deformation energy of an eigenmode with a normalized amplitude on the undulating membrane M with surface area 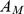 is then given by


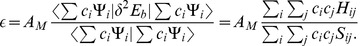
(8)

For instance, the element 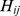 for the Laplace deformation energy writes in local coordinates 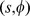 as


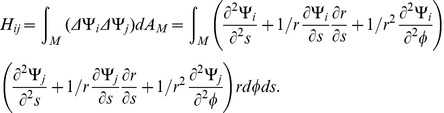
(9)

One must evaluate the matrix H for each azimuthal number m, the indice was omitted for simplicity. The vector 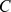 of basis function coefficients 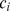 for the eigenmode and the minimized deformation energy 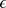 are then found by solving the eigenvalue problem 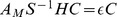. Eigenmodes with the lowest (or second lowest) eigenvalues are considered in each azimuthal number class when comparing with the measured PCA modes.

### Comparison of theoretical mean square fluctuation amplitude and PCA mode variances

An amplitude 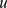 of a deformation function 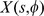 on the undulating membrane M is evaluated as


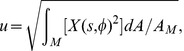
(10)

where 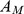 is the surface of the undulating membrane. Bending deformation energies 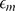 are evaluated for energy eigenmodes 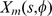 normalized in the sense of Eq. [10]. Similarly, experimental PCA modes 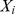 are normalized with respect to the surface area of the ROI 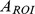 (cf. Fig. 1D). Because DHM cannot assess the CMF on the whole fluctuating membrane surface, but only in a restricted ROI, one must introduce a correction factor when relating the theoretically calculated mean square fluctuation amplitudes 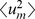 to the measured PCA mode variances 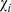. This factor for an eigenmode 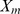 is given by


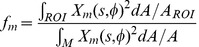
(11)

such that the theoretically expected variance as captured by a PCA mode is


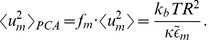
(12)

## Supporting Information

Text S1 **Bending elasticity and equilibrium shape.**
(PDF)

Table S1
**Bending deformation energies** 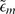 **for eigenmodes depicted in** **Figures 3** **and S3.** The evaluated values 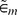 used for the estimation of the expected PCA mode variances in equation 10 are shown in the the Table S1.(PDF)

Figure S1
**Higher order PCA modes from the discocyte RBC group of n = 198 cells.** For the higher order (lower energy) PCA modes one observes that the spatial pattern of the components is less influenced by the geometry of the cell as the PCA mode variance decreases. Planar waves seem therefore to be a good decomposition basis of the membrane fluctuations for spatial frequencies 
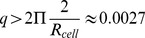
 rad/nm.(EPS)

Figure S2
**ATP depletion/Decomposition of membrane fluctuation onto planar modes.** (A) Mean fluctuation amplitudes 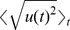 of individual cells averaged in the ROI after t = 0,1,2,4 h of incubation in depletion medium for n = 198,43,47,36 cells. After 4 hours of ATP depletion, about half of the cells showed clearly reduced fluctuation amplitudes. These cells showed strongly reduced coherent fluctuations in the PCA approach. (B) Mean square fluctation amplitudes of planar modes 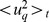 in function of spatial wavevector 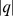, n = 198 cells, error bars denote SD-Value. Measured mean square fluctuation amplitudes 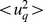 were normalized by theoretically expected amplitudes of bending modes 
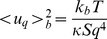
. The value for the bending modulus 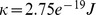 was measured based on PCA modes in the long wavelength regime. Independently of intracellular ATP concentration, an increase of fluctuation amplitudes with respect to pure bending modes are observed for spatial wavevectors 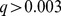 rad/nm. For fluctuations at high spatial frequencies, the standard deviation around the measured average fluctuation amplitudes (n = 198 cells) increases considerably. This can partially be explained by experimental noise: DHM and other quantitative phase imaging methods are confronted with noise at higher spatial frequencies that also contributes to the measured mean square fluctuation amplitudes at high spatial wavevectors. Therefore, we think that the measurements must be taken with precaution for 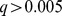.(EPS)

Figure S3 **Spontaneous curvature (SC) bending deformation eigenmodes.**
(EPS)

Figure S4
**Experimental and theoretical CMF amplitude distribution on RBC surface/Visualisation of ROI used for PCA and theoretical model.** (A) Mean CMF amplitudes 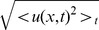 measured in a group of n = 198 cells (green line) along average profile of RBC surface (blue line). The measured amplitudes have been corrected by a factor 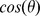, where 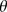 is the angle that the surface normal makes with the z-axis. Black vertical lines indicate the boundaries of the ROI that was used for PCA analysis. (B) Equilibrium shape of model cell 1 (straight line) and theoretical CMF amplitude distribution (dashed line) obtained by adding the mean squared fluctuation amplitudes of the first 9 eigenmodes depicted in Figure 3B. Amplitude is overestimated for better visibility. Black vertical lines indicate the boundaries of the theoretical eigenmodes depicted in Figure 3B,C. (C) Theoretical CMF amplitudes (green line) along profile of RBC equilibrium shape (blue line). Amplitudes have been corrected by a factor 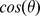. This representation of the theoretical results is in good agreement with experimental data in (A).(EPS)

Figure S5
**Influence of equilibrium shape and boundary conditions on Laplace deformation energy eigenmodes.** (A) Equilibrium shape of model cell 2 (straigth line) and theoretical CMF amplitude distribution (dashed line) obtained by adding the mean squared fluctuation amplitudes of the first 9 eigenmodes depicted in Figure S5C. CMF Amplitude is overestimated for better visibility. Black vertical lines indicate the boundaries of the theoretical eigenmodes depicted in Figure S5C (B) Equilibrium shape of model cell 3 (straight line) and theoretical CMF amplitude distribution (dashed line) obtained by adding the mean squared fluctuation amplitudes of the first 9 eigenmodes depicted in Figure S5D. Black vertical lines indicate the boundaries of the theoretical eigenmodes depicted in Figure S5D. (C) Eigenmodes that minimize Laplace deformation energy shown in the ROI for model cell 2. Eigenmodes are plotted as functions 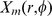 (D) Eigenmodes that minimize Laplace deformation energy shown in the ROI for model cell 3. (C) Comparison of theoretical and measured fluctuation amplitudes of PCA modes.(EPS)
