# Supplementary material for: Spatially-Resolved Eigenmode Decomposition of Red Blood Cells Membrane Fluctuations Questions the Role of ATP in Flickering
Source: PLoS One. 2012 Aug 10;7(8):e40667. doi: 10.1371/journal.pone.0040667 (PMC3416845; doi:10.1371/journal.pone.0040667)
Supplement: Text S1 — Bending elasticity and equilibrium shape. (PDF) [file pone.0040667.s001.pdf]

Supporting Text S1: Bending elasticity and equilibrium shape

In the framework of the spontaneous curvature bending elasticity, a cell with a given surface A and volume V will take the shape that minimizes the bending energy E_b

$$E_b = \frac{\kappa}{2} \int_M (C_1 + C_2 - C_0)^2 \, dA, \tag{1}$$

where C_1 and C_2 denote the local principal curvatures on the undulating membrane surface M, respectively, and where C_0 is the spontaneous curvature of the bilayer. The spontaneous curvature is thought to arise from the asymmetric composition of the membrane layers. In the Bilayer Coupling Hypothesis, the influence of the two asymmetric monolayers is introduced by requiring that the surface area difference of the monolayers ΔA is given and does not change in a deformation.

The equilibrium shape that is used for calculations and depicted in Figure 2 of the article is derived using the approach found in Seifert et al. [23]. The shape equations for a cell with volume V and surface A are obtained by minimizing the bending energy. For the spontaneous curvature model this writes

$$\delta \left(\int_M (C_1 + C_2 - C_0)^2 \, dA + PV + \Sigma A \right) = 0 \tag{2}$$

Here P and Σ are lagrangian multipliers that regulate constant surface area A and volume V. In the Bilayer Coupling Hypothesis, the requirement of a constant surface area difference ΔA of the monolayers is mathematically introduced with an additional Lagrangian multiplier for the mean curvature $\langle (C_1 + C_2)/2 \rangle$. Because of formal equivalence, both energies give rise to the same stationary shapes. The shape equations derived from 2 are:

$$\begin{aligned}
\frac{\partial \theta}{\partial s} &= U \\
\frac{\partial U}{\partial s} &= -\frac{U}{r}\cos\theta + \frac{\cos\theta \sin\theta}{r^2} + \frac{\gamma}{r}\sin\theta + \frac{Pr}{2}\cos\theta \\
\frac{\partial \gamma}{\partial s} &= (U - C_0)^2/2 - \frac{\sin\theta^2}{2r^2} + Pr\sin\theta + \Sigma \\
\frac{\partial r}{\partial s} &= \cos\theta
\end{aligned} . \tag{3}$$

Here $\theta(s)$ is the angle that the surface normal makes with the vertical z-axis, r[s] is the distance from z-axis and $\gamma[s]$ is a Lagrange parameter function. Boundary conditions and values for Σ, P and C_0 fix the equilibrium shape of the cell. For model cell 1 depicted in Figure 2 these are:

$$P = 29.8718,\ \Sigma = -0.7P^{2/3},\ C_0 = 0,\ U(0) = -0.8P^{1/3},\ \theta(0) = 0,\ r(0) = 0,\ \gamma(0) = 0. \tag{4}$$

For model cell 2 depicted in Figure S5A:

$$P = 32.7680,\ \Sigma = -0.65P^{2/3},\ C_0 = 0,\ U(0) = -1.2P^{1/3},\ \theta(0) = 0,\ r(0) = 0,\ \gamma(0) = 0. \tag{5}$$

For modell cell 3 depicted in Figure S5B:

$$P = 23.8879,\ \Sigma = -0.7P^{2/3},\ C_0 = 0,\ U(0) = -0.8P^{1/3},\ \theta(0) = 0,\ r(0) = 0,\ \gamma(0) = 0. \tag{6}$$

The cell shape can then be expressed as a curve $C(s) = [r(s)\ z(s)]$ revolved around the z-axis. With the parameter P the length scale of the curve can be determined. For model cell 1 and 2, it is chosen such that s runs from s=0 on the z-axis to $s = \frac{\pi}{2}$ where the curve is aligned with the horizontal, i.e. $\frac{\partial z(s)}{\partial s} = 0$. For model cell 3, the endpoint of the curve is not aligned with the horizontal in order to evaluate the influence of the imposed boundary constraints on the energy eigenmodes of deformation.
