# Supplementary material for: Spatially-Resolved Eigenmode Decomposition of Red Blood Cells Membrane Fluctuations Questions the Role of ATP in Flickering
Source: PLoS One. 2012 Aug 10;7(8):e40667. doi: 10.1371/journal.pone.0040667 (PMC3416845; doi:10.1371/journal.pone.0040667)
Supplement: Table S1 — Bending deformation energies for eigenmodes depicted in Figures 3 and S3. The evaluated values used for the estimation of the expected PCA mode variances in equation 10 are shown in the the Table S1. (PDF) [file pone.0040667.s002.pdf]

Mode number	1	2	3	4	5	6	7	8	9
Laplace $\widetilde{\epsilon_m}$	145	430	1075	1507	1576	2401	3347	4899	10907
BCH $\widetilde{\epsilon_m}$	151	338	900	928	882	2176	2378	4691	7464
SC $\widetilde{\epsilon_m}$	164	225	451	347	-30	1024	1047	2243	3088
