# Supplementary figures and images for: Spatially-Resolved Eigenmode Decomposition of Red Blood Cells Membrane Fluctuations Questions the Role of ATP in Flickering
Source: PLoS One. 2012 Aug 10;7(8):e40667. doi: 10.1371/journal.pone.0040667 (PMC3416845; doi:10.1371/journal.pone.0040667)

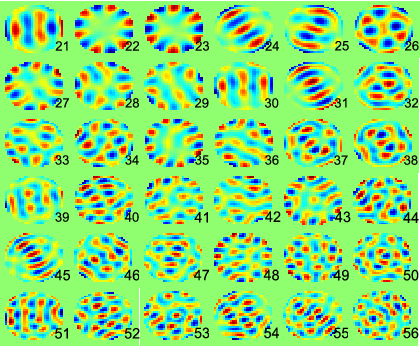

Supplement: Figure S1 — Higher order PCA modes from the discocyte RBC group of n = 198 cells. For the higher order (lower energy) PCA modes one observes that the spatial pattern of the components is less influenced by the geometry of the cell as the PCA mode variance decreases. Planar waves seem therefore to be a good decomposition basis of the membrane fluctuations for spatial frequencies rad/nm. (EPS) [file pone.0040667.s003.tif]

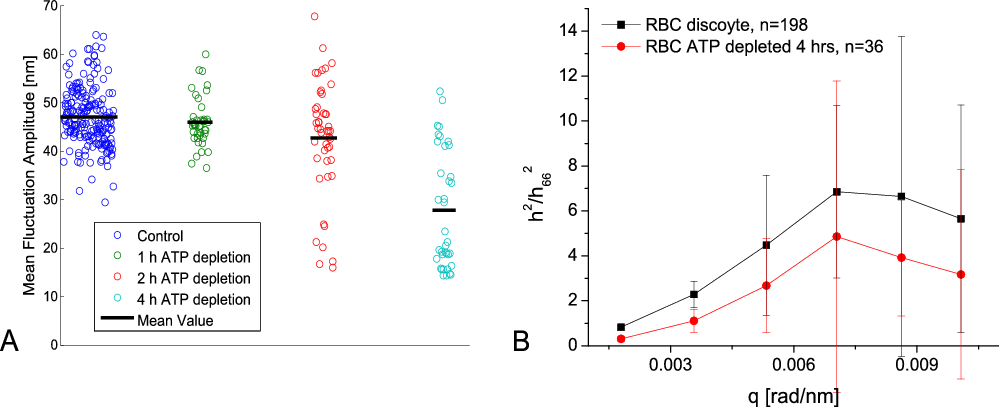

Supplement: Figure S2 — ATP depletion/Decomposition of membrane fluctuation onto planar modes. (A) Mean fluctuation amplitudes of individual cells averaged in the ROI after t = 0,1,2,4 h of incubation in depletion medium for n = 198,43,47,36 cells. After 4 hours of ATP depletion, about half of the cells showed clearly reduced fluctuation amplitudes. These cells showed strongly reduced coherent fluctuations in the PCA approach. (B) Mean square fluctation amplitudes of planar modes in function of spatial wavevector , n = 198 cells, error bars denote SD-Value. Measured mean square fluctuation amplitudes were normalized by theoretically expected amplitudes of bending modes . The value for the bending modulus was measured based on PCA modes in the long wavelength regime. Independently of intracellular ATP concentration, an increase of fluctuation amplitudes with respect to pure bending modes are observed for spatial wavevectors rad/nm. For fluctuations at high spatial frequencies, the standard deviation around the measured average fluctuation amplitudes (n = 198 cells) increases considerably. This can partially be explained by experimental noise: DHM and other quantitative phase imaging methods are confronted with noise at higher spatial frequencies that also contributes to the measured mean square fluctuation amplitudes at high spatial wavevectors. Therefore, we think that the measurements must be taken with precaution for . (EPS) [file pone.0040667.s004.tif]

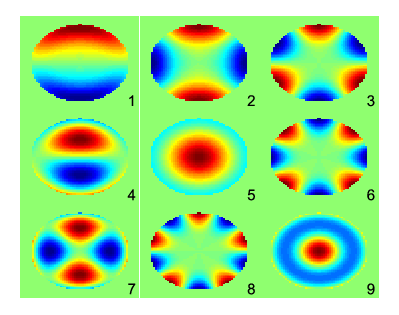

Supplement: Figure S3 — Spontaneous curvature (SC) bending deformation eigenmodes. (EPS) [file pone.0040667.s005.tif]

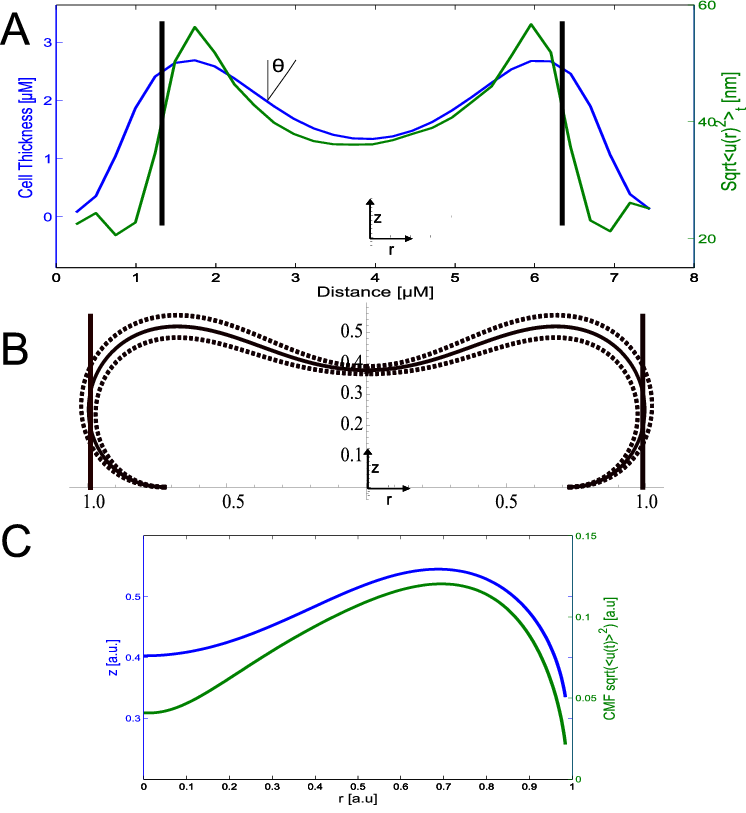

Supplement: Figure S4 — Experimental and theoretical CMF amplitude distribution on RBC surface/Visualisation of ROI used for PCA and theoretical model. (A) Mean CMF amplitudes measured in a group of n = 198 cells (green line) along average profile of RBC surface (blue line). The measured amplitudes have been corrected by a factor , where is the angle that the surface normal makes with the z-axis. Black vertical lines indicate the boundaries of the ROI that was used for PCA analysis. (B) Equilibrium shape of model cell 1 (straight line) and theoretical CMF amplitude distribution (dashed line) obtained by adding the mean squared fluctuation amplitudes of the first 9 eigenmodes depicted in Figure 3B. Amplitude is overestimated for better visibility. Black vertical lines indicate the boundaries of the theoretical eigenmodes depicted in Figure 3B,C. (C) Theoretical CMF amplitudes (green line) along profile of RBC equilibrium shape (blue line). Amplitudes have been corrected by a factor . This representation of the theoretical results is in good agreement with experimental data in (A). (EPS) [file pone.0040667.s006.tif]

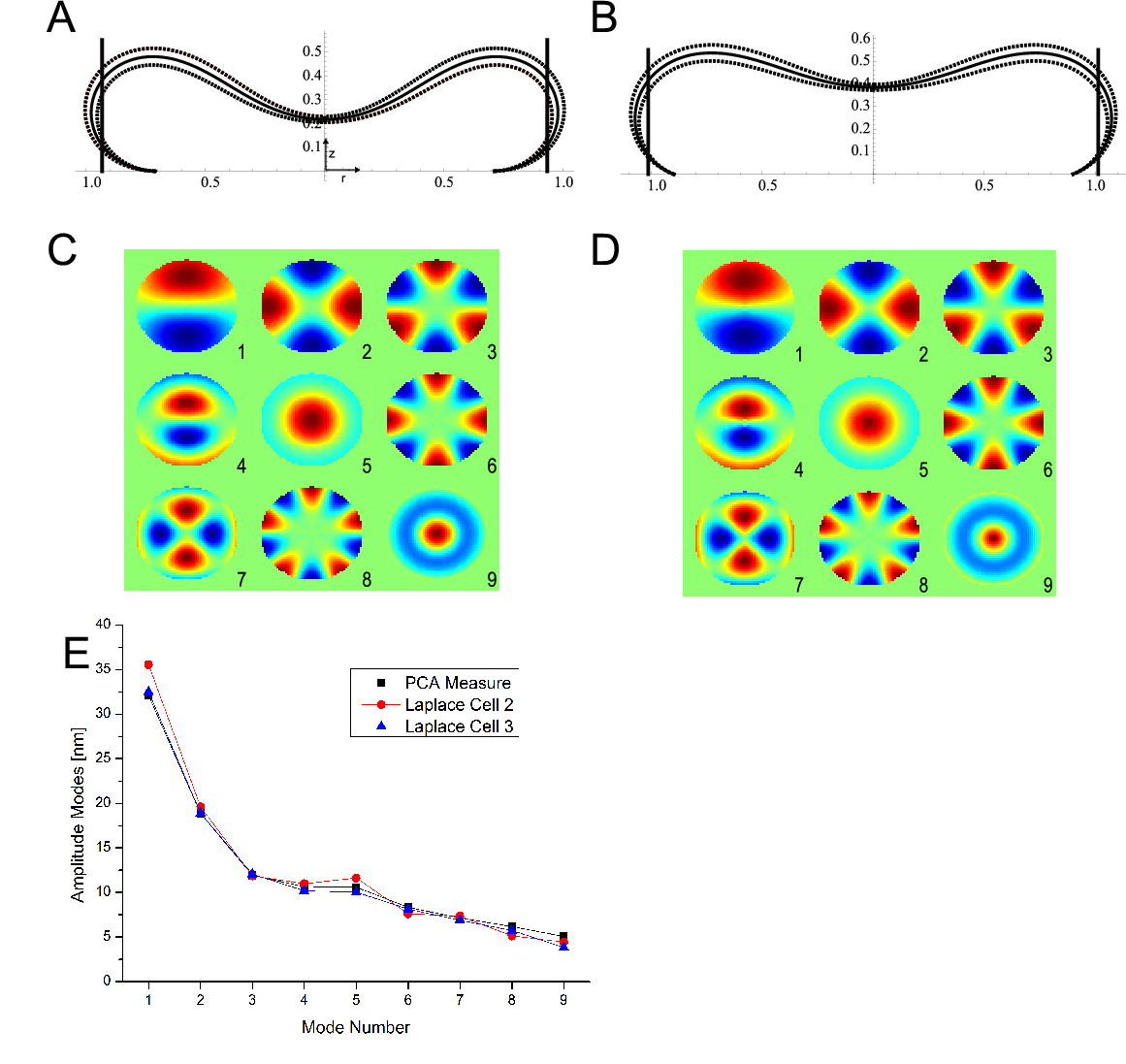

Supplement: Figure S5 — Influence of equilibrium shape and boundary conditions on Laplace deformation energy eigenmodes. (A) Equilibrium shape of model cell 2 (straigth line) and theoretical CMF amplitude distribution (dashed line) obtained by adding the mean squared fluctuation amplitudes of the first 9 eigenmodes depicted in Figure S5C. CMF Amplitude is overestimated for better visibility. Black vertical lines indicate the boundaries of the theoretical eigenmodes depicted in Figure S5C (B) Equilibrium shape of model cell 3 (straight line) and theoretical CMF amplitude distribution (dashed line) obtained by adding the mean squared fluctuation amplitudes of the first 9 eigenmodes depicted in Figure S5D. Black vertical lines indicate the boundaries of the theoretical eigenmodes depicted in Figure S5D. (C) Eigenmodes that minimize Laplace deformation energy shown in the ROI for model cell 2. Eigenmodes are plotted as functions (D) Eigenmodes that minimize Laplace deformation energy shown in the ROI for model cell 3. (C) Comparison of theoretical and measured fluctuation amplitudes of PCA modes. (EPS) [file pone.0040667.s007.tif]
